# Modeling Surface Disinfection Needs To Meet Microbial Risk Reduction Targets

**DOI:** 10.1128/AEM.00709-18

**Published:** 2018-08-31

**Authors:** Amanda M. Wilson, Kelly A. Reynolds, Jonathan D. Sexton, Robert A. Canales

**Affiliations:** a Mel and Enid Zuckerman College of Public Health, University of Arizona, Tucson, Arizona, USA; University of Helsinki

**Keywords:** MS2, fomite, infection control, quantitative microbial risk assessment, risk target

## Abstract

Nosocomial viral infections are an important cause of health care-acquired infections where fomites have a role in transmission. Using stochastic modeling to quantify the effects of surface disinfection practices on nosocomial pathogen exposures and infection risk can inform cleaning practices. The purpose of this study was to predict the effect of surface disinfection on viral infection risks and to determine needed viral reductions to achieve risk targets. Rotavirus, rhinovirus, and influenza A virus infection risks for two cases were modeled. Case 1 utilized a single fomite contact approach, while case 2 assumed 6 h of contact activities. A 94.1% viral reduction on surfaces and hands was measured following a single cleaning round using an Environmental Protection Agency (EPA)-registered disinfectant in an urgent care facility. This value was used to model the effect of a surface disinfection intervention on infection risk. Risk reductions for other surface-cleaning efficacies were also simulated. Surface reductions required to achieve risk probability targets were estimated. Under case 1 conditions, a 94.1% reduction in virus surface concentration reduced infection risks by 94.1%. Under case 2 conditions, a 94.1% reduction on surfaces resulted in median viral infection risks being reduced by 92.96 to 94.1% and an influenza A virus infection risk below one in a million. Surface concentration in the equations was highly correlated with dose and infection risk outputs. For rotavirus and rhinovirus, a >99.99% viral surface reduction would be needed to achieve a one-in-a-million risk target. This study quantifies reductions of infection risk relative to surface disinfectant use and demonstrates that risk targets for low-infectious-dose organisms may be more challenging to achieve.

**IMPORTANCE** It is known that the use of EPA-registered surface disinfectant sprays can reduce infection risk if used according to the manufacturer's instructions. However, there are currently no standards for health care environments related to contamination levels on surfaces. The significance of this research is in quantifying needed reductions to meet various risk targets using realistic viral concentrations on surfaces for health care environments. This research informs the design of cleaning protocols by demonstrating that multiple applications may be needed to reduce risk and by highlighting a need for more models exploring the relationship among microbial contamination of surfaces, patient and health care worker behaviors, and infection risks.

## Abstract (precis)

It is known that the use of EPA-registered surface disinfectant sprays can reduce infection risk if used according to the manufacturer's instructions. However, there are currently no standards for health care environments related to contamination levels on surfaces. The significance of this research is in quantifying needed reductions to meet various risk targets using realistic viral concentrations on surfaces for health care environments. This research informs the design of cleaning protocols by demonstrating that multiple applications may be needed to reduce risk and by highlighting a need for more models exploring the relationship among microbial contamination of surfaces, patient and health care worker behaviors, and infection risks.

## INTRODUCTION

Viruses account for a large portion of acquired infectious diseases in indoor environments, including hospitals (1, 2). Enteric viruses, such as rotavirus, and respiratory viruses, such as rhinovirus and influenza virus, continue to contribute to global disease burdens. Although there are licensed rotavirus vaccines available in the United States, rotavirus illness remains an important cause of diarrhea mortality for young children (3). Rhinoviruses account for 20 to 40% of cases of the common cold and can lead to further health issues, including sinusitis, asthma, otitis media, and pneumonia (4). Although the mortality rates for influenza and pneumonia vary from year to year, the burden of disease remains high (5). The CDC estimates that 9.2 million to 60.8 million influenza cases occur each year in the United States (https://www.cdc.gov/flu/about/disease/2015-16.htm).

Viruses spread rapidly in indoor environments, and it is often challenging to monitor viral nosocomial disease, resulting in data that may underestimate the true effect of viral pathogens in health care settings (1, 4). As molecular methods for detecting viral pathogens improve, monitoring viral disease is becoming less expensive and therefore more feasible (6). Improved detection methodology has raised awareness of the role of viruses in respiratory and gastrointestinal nosocomial diseases (6). Although hand washing and isolation of infectious patients have been recognized strategies in controlling the spread of viruses, more recently, attention has been given to the role of inanimate objects (i.e., fomites) in pathogen spread. Surfaces in health care settings act as vehicles and reservoirs for pathogens. People may come into contact with surfaces and either become contaminated or contaminate the surface, and aerosolized microbes may be deposited on surfaces (7). Some surfaces, such as rolling equipment or shoes, are moved from one room to another, leading to contamination of the equipment or contamination of the environment into which they are moved. Acknowledging the role of fomites in disease transmission has raised awareness of surface-cleaning protocols and their ability to reduce the pathogen load in health care environments (1). Although there are many routes of exposure to health care-associated infection (HAI)-causing pathogens, the role of environmental contamination in HAI-causing-pathogen exposure is thought to be underestimated (8). Evidence of the contribution of environmental contamination to nosocomial disease transmission includes model estimates, epidemiological studies, intervention studies, and clinical trials (9–11). Hand washing before and after patient visits remains important, but high compliance rates can be difficult to maintain, making the use of surface disinfectants an important component of infection control protocols (6).

A single wipe with a wet or soapy cloth can reduce surface concentrations of both viral and bacterial pathogens by more than 1 log_10_ unit (12). However, this reduction may not be enough to address residual pathogens on surfaces (12). If pathogens are not removed, they can persist for hours to days on surfaces, resulting in further spread and transmission of disease (7). Some viruses, such as rhinovirus, which may remain viable for 1 to 3 h (13), also survive well on skin.

Larger reductions in surface concentrations can reduce opportunities for viruses to persist and participate in future cross-contamination and transmission events, and it has been demonstrated that the products used during cleaning and the thoroughness of cleaning may have greater impacts on the microbial surface burden than cleaning frequency (14). Quantitative microbial risk assessment (QMRA) models have predicted that a 2-log_10_-unit bacterial reduction on fomites could reduce infection risk from a single fomite contact to less than 1/1,000,000 (15). For viruses, the log unit reductions needed to decrease infection risk may be greater than that of bacterial pathogens, due to lower infectious doses. In the same QMRA model, a 3.44-log_10_-unit reduction was needed to reduce norovirus infection risk to 1/1,000,000 (15). For some viruses, doses of less than 1 50% tissue culture infective dose (TCID_50_) can infect 50% of an exposed population (16). Low infectious doses underscore the importance of regular and effective cleaning or consistent application of surface disinfectants to address pathogenic-virus loads on fomites.

In addition to increasing surface disinfection and encouraging hand hygiene compliance, there is growing interest in understanding the movement of viral spread in health care settings. Using harmless bacteriophages as surrogates for viral pathogens has been a popular approach, and this methodology has been used in a variety of environments and contexts, including offices, emergency medical vehicles, long-term health care facilities, and pediatric and neonatal intensive care unit patient rooms (17–20). Seeding a commonly touched surface and then swabbing a variety of high-touch surfaces and hands after a given period of time characterizes the fate of viruses in the environment and can elucidate which surfaces should be prioritized in cleaning protocols. Sexton et al. used MS2 bacteriophage as a tracer representing enteric viruses and found that the phage quickly spread from inoculated surfaces in a long-term-care wing into an adjacent independent/assisted-living wing of one long-term-care facility within a few hours during the staff's routine practice of care (21). Repeating trials in bacteriophage tracer studies in which interventions are implemented may evaluate how those interventions reduce spread over the course of a day. The use of bacteriophage tracer results to better characterize and anticipate the viral movement in real-world environments through modeling is a developing area of research and a novel approach in investigating intervention efficacy. Translating reductions observed with bacteriophage tracers in real-world environments into predicted infection risk reductions will better inform hygiene practices and cleaning routines than efficacy data gathered in laboratory settings.

This study predicts the doses of rotavirus, rhinovirus, and influenza A virus and their respective infection risks after single (case 1) and multiple (case 2) fomite and orifice contacts under various surface disinfection reduction conditions. Other viral surface reductions were explored in order to quantify the reductions required to meet infection risk targets. Rotavirus was included because it has been used to represent enteric viruses as a conservative approach in quantitative microbial risk assessments, due to its low infectious dose (22, 23). Rhinovirus was a virus of interest due to its low infectious dose and its ability to infect a host from hand-to-eye and hand-to-nose contacts (16). Influenza A virus was included due to its ability to cause infection from hand-to-eye, -nose, and -mouth contacts, and its infectious dose (9.45 × 10^5^), according to the Quantitative Microbial Risk Assessment (QMRA) Wiki, is multiple orders of magnitude greater than those of rotavirus and rhinovirus [http://qmrawiki.canr.msu.edu/index.php/Quantitative_Microbial_Risk_Assessment_(QMRA)_Wiki]. The inclusion of these three viruses allowed evaluation of the effects of infectious dose and route of infection on estimated infection risks and intervention efficacies.

## RESULTS

A 94.1% virus concentration reduction on surfaces resulted in a 94.1% median dose reduction for rotavirus, rhinovirus, and influenza A virus in both modeled cases. Case 1 rotavirus, rhinovirus, and influenza A virus median infection risks were reduced by 94.1% for all three viruses, and case 2 median infection risks were reduced by 92.96% to 94.1% (Table 1). High correlation coefficients between surface concentration and dose and infection risk explained why a 94.1% reduction in the viral concentration resulted in a 94.1% reduction in risk in case 1. Case 1 and case 2 correlation coefficients for viral surface concentrations and dose and for viral surface concentration and infection risks under intervention conditions were 0.83 to 0.84 and 0.57 to 0.69, respectively. Case 1 infection risk reductions in the 95th percentiles for rotavirus, rhinovirus, and influenza A virus ranged from 92.88 to 94.1%, while for case 2, they ranged from 45.51 to 94.10% (Table 1).

**TABLE 1:** Percent reduction in infection risk from a 94.1% reduction in viruses on surfaces for case 2 (6 h of contact activity)

Virus	% reduction in infection risk	
	Median	95th percentile
Rotavirus	92.96	50.24
Rhinovirus	93.65	45.51
Influenza A virus	94.10	94.10

Infection risks for single fomite contacts were less than those for 6 h of contact activity. Case 1 median predicted infection risks, without a 94.1% reduction on surfaces, ranged from 4.57 × 10^−4^ (rotavirus) to 2.56 × 10^−10^ (influenza A virus) (Table 2). These predicted risks are less than that predicted for norovirus infection (probability of infection = 2.7 × 10^−3^) in other quantitative microbial risk assessments (15). Case 2 median baseline infection risks ranged from 7.24 × 10^−2^ (rotavirus) to 3.43 × 10^−7^ (influenza A virus), 2 to 3 log_10_ units higher than infection risks from single contacts (Table 2).

**TABLE 2:** Median probabilities of infection from case 1 (single fomite contact) and case 2 (6 h of contact activity) under baseline and intervention conditions

Virus	Case 1		Case 2	
	Baseline	Intervention	Baseline	Intervention
Rotavirus	4.57 × 10^−4^	2.70 × 10^−5^	7.24 × 10^−2^	5.10 × 10^−3^
Rhinovirus	4.86 × 10^−4^	2.87 × 10^−5^	2.78 × 10^−2^	1.77 × 10^−3^
Influenza A virus	2.56 × 10^−10^	1.51 × 10^−11^	3.43 × 10^−7^	2.02 × 10^−8^

Under case 2 conditions, 94.1% reduction in virus concentrations on surfaces reduced influenza A virus risk well below one in a million (probability of infection = 2.02 × 10^−8^). However, for rotavirus and rhinovirus, infection risks were both on the order of one in a thousand. A 99.999% reduction, one of the highest current efficacy claims for disinfection products, decreased the risk of infection for rotavirus and rhinovirus to 9.48 × 10^−7^ and 3.05 × 10^−7^, respectively, placing risk below one in a million (Tables 3 and 4).

**TABLE 3:** Impact of percent reduction in concentration of rotavirus, rhinovirus, and influenza A virus on risk, assuming case 2 conditions

% reduction in viral concn	Infection risk		
	Rotavirus	Rhinovirus	Influenza A virus
94.1	5.10 × 10^−3^	1.77 × 10^−3^	2.02 × 10^−8^
99	9.46 × 10^−4^	3.09 × 10^−4^	3.64 × 10^−9^
99.9	9.24 × 10^−5^	3.19 × 10^−5^	3.51 × 10^−10^
99.99	9.66 × 10^−6^	3.09 × 10^−6^	3.79 × 10^−11^
99.999	9.48 × 10^−7^	3.05 × 10^−7^	3.58 × 10^−12^

**TABLE 4:** Percent reduction required for median probabilities of viral infection to meet risk levels, assuming case 2 conditions

Risk level	Required % reduction on surfaces		
	Rotavirus	Rhinovirus	Influenza A virus^*a*^
1/1000	98.87812	96.79529	None
1/10,000	99.89373	99.67195	None
1/100,000	99.98879	99.96747	None
1/1,000,000	99.99892	99.99691	None

a Baseline risk levels for influenza A virus were below 1/1,000,000. None, no further reduction would be needed beyond the observed 94.1% to meet these risk thresholds.

## DISCUSSION

The overall high reductions in infection risks support surface disinfection prioritization in cleaning protocols. The difference in risk reduction from a single fomite contact to 6 h of contact activity underscores the fact that longer exposure times diminish the risk mitigation offered by surface disinfection, especially for pathogens that may have low infectious doses, such as rotavirus and rhinovirus. In environments with viral concentrations higher than those modeled in this study, the diminishing effect of surface disinfection over time may be even more apparent, as increased pathogen doses yield higher risks of infection. The decrease in percent reductions seen for the 95th percentile from 1 s of contact to 6 h of contact demonstrates that those who touch their eyes, nose, mouth, or fomites more often than what is considered “average” experience less beneficial effect from a single surface cleaning than those who have average exposures. This supports the reapplication of surface disinfection so that higher percent reductions are met to achieve the same reductions in infection risk, especially for those who may be more exposed than the average patient or health care staff member.

High and consistent reductions in influenza A virus infection risk for this study scenario demonstrate that the dose-response curve for a pathogen can affect the observed intervention efficacy. Depending upon the baseline modeled dose, an equal change in dose at any point along the curve may not have an equal effect on infection risk, depending upon the shape of the dose-response curve. This means that the pathogen reductions on surfaces needed to meet particular risk targets may vary from one pathogen to another. For example, Ryan et al. (15) estimated that a 99.2% reduction would be needed to meet a one-in-a-million risk target for Escherichia coli O157:H7, while norovirus required a 99.96% reduction. It was also estimated that other bacteria, such as Pseudomonas and staphylococci, had associated single-touch risks that already met the one-in-a-million target risk (15). A current challenge to understanding surface disinfection efficacy in risk target terms is a lack of consensus about what acceptable risk means in health care environments. Models that quantify the pathogen reductions needed to achieve risk targets, such as in this study and that of Ryan et al. (15), will help inform conversations aimed at developing future standards for health care cleanliness. However, more environmental sampling is needed to further inform these models and improve their relevance in decision making. A limitation of this study was that assumptions were made concerning concentrations of viral pathogens on surfaces based on one study (24) in which data related to rhinovirus and influenza A virus were not incorporated. To our knowledge, measured concentrations of rhinovirus and influenza A virus on health care fomites were not available to be utilized in informing distributions for viral concentrations on surfaces. More data characterizing concentrations of pathogens on commonly touched surfaces with reported limits of detection and proportions of samples above or below the limits of detection would result in more informed assumptions regarding the concentrations of pathogens in health care environments. Additionally, ratios of detected to viable virus for health care surface samples would further inform assumed concentrations of viable viruses on surfaces. Ratios of detected to viable virus in this study were only specific to enteric viruses, and some values used to inform the distribution of ratios were not specific to rotavirus detection or quantification. However, data were not available to inform the distribution of these ratios for rhinovirus and influenza A virus. Having more data to inform these ratios would diminish current uncertainty in translating quantitative PCR (qPCR) data to estimate concentrations of viable pathogens on health care surfaces assumed in QMRA modeling.

In addition to contact with fomites, other exposure pathways may contribute to infection risk, such as hand-to-hand and hand-to-patient contacts and inhalation of aerosolized pathogens. To understand the role of surface disinfection in reducing the doses from indirect exposures, more complex models are needed to address the relationships between surface disinfection, fomite contacts, and patient-to-patient or health care worker-to-patient contacts and the influence of hand hygiene practices. To improve upon the accuracy of QMRA modeling as a method to explore the efficacy of interventions, understanding the mechanisms by which cross-contamination occurs at the microactivity level would allow more accurate portrayals of viral accumulation on the hands. The equations used in this study assume 1 s of contact or an equilibrium value on hands. Minute-by-minute changes in virus concentrations on surfaces and hands are not accounted for. There may be moments that allow high exposures between surface disinfection cleanings that are not currently represented due the steady-state assumption.

Some models aim to capture health care worker and patient behaviors to understand the minute-by-minute transfer of pathogens and the effects of various hygiene interventions, but more models are needed to address the vast number of environments, pathogenic exposures, and scenarios that may occur within health care settings (25, 26). Accurate microlevel activity data for various health care professionals are extremely limited, and the effect of contact duration and repetitive contacts on the transfer efficiencies for microbes is relatively unknown. Contact duration and repetitive contacts have been shown to affect transfer efficiencies for chemical exposures (27, 28). If this is also true for microbial exposures, it is possible that models estimating doses that assume a transfer efficiency unrelated to contact behavior do not portray the movement of viruses during contact events appropriately. More information regarding duration and contact frequency with a variety of surface types would improve health care transmission models and allow more complex and accurate capture of pathogen movement in health care settings.

Data regarding concentrations of viable pathogens in health care environments are also lacking, especially for viral pathogens. With more accurate distributions of pathogens in health care environments, more certainty could be placed in QMRAs and the intervention protocols they support. Models assuming a single value for concentrations on surfaces and hands over time, such as the one in this study, may be helpful but may not represent what is actually happening at higher time resolution in the patient care environment. Accounting for small incremental changes in concentrations over time on surfaces and hands combined with real-world distributions of viable pathogen concentrations on health care surfaces would allow more accurate predictions of intervention effects, compliance with cleaning protocols, and the necessary frequency of cleaning routines. This could lead to a more accurate characterization of interventions and their effects in real-world environments. QMRA infection risk and intervention efficacy estimates, in conjunction with relevant epidemiological studies to directly link interventions with observed health outcomes, would benefit infection control professionals by informing current cleaning practices. It would additionally inform stakeholders about the frequency and duration of cleaning rounds needed to achieve lasting claimed reductions in health care environments.

## MATERIALS AND METHODS

### Creating a distribution for virus concentrations on health care surfaces.

Data (i.e., minimum, maximum, limit of detection, and proportion below the limit of detection) for rotavirus concentrations on health care surfaces reported in the literature were used to create a distribution of rotavirus, rhinovirus, and influenza A virus concentrations for modeled health care surfaces (24). The portion of surface concentrations reported as below the assay limit of detection by Ganime et al. (24) was assumed to be uniformly distributed. The portion above the limit of detection reported by Ganime et al. (24) was assumed to be an exponential decay, where there is a relatively low probability of very high values. To mimic reported data, values above and below the limit of detection, 3.4 genome copies/ml, were sampled so that the fractions of samples above and below the limit of detection were reflective of those reported by Ganime et al. (24). Using the inverse cumulative distribution function, the 99th percentile (assumed to be equivalent to the maximum value, 2.94 × 10^3^ genome copies/ml, from Ganime et al. [24]) was used to compute the rate parameter of the exponential distribution.

Per iteration, a surface concentration was randomly sampled from a distribution intended to capture concentrations both below and above the limits of detection. Surface concentrations were obtained by qPCR methods. To address viability, a uniform distribution of proportions of viable viruses per milliliter to genome copies per milliliter ranging from 1.533 × 10^−5^ to 1.542 × 10^−1^ was used. These proportions originated from ratios of qPCR and culture method results for enterovirus concentrations in water samples that had undergone various treatment steps (29). These ratios were thought to be acceptable within the context of health care fomites, assuming that fomites are regularly cleaned, because there is likely to be RNA on a surface that is unassociated with a viable organism. However, it is acknowledged that this assumption contributes a large amount of uncertainty in relating the qPCR concentrations to assumed viable virus concentrations. By applying ratios of viable viral particles to genome copies defined by Francy et al. (29) to distributions defined by Ganime et al. (24) and assuming 1 ml translates into 100 cm^2^, units were converted to viruses per square centimeter. To apply a percent reduction in the viral concentration on a surface due to surface disinfection, “1 minus reduction fraction” was multiplied by the viral concentrations. The effect of a single round of cleaning with an Environmental Protection Agency (EPA)-registered surface disinfectant was modeled using a viral reduction observed in a viral tracer study conducted in an urgent care facility in which a 94.1% reduction in virus concentrations on surfaces and hands was measured (K. A. Reynolds, J. Sexton, T. Pivo, K. Humphrey, R. A. Leslie, and C. P. Gerba, submitted for publication).

### Case 1: modeling infection risk from a single fomite contact.

Case 1 comprised a single hand-to-fomite contact followed by a single hand-to-orifice contact, mimicking the process performed in the Ryan et al. (15) QMRA study. For the initial hand-to-fomite contact, the fomite was modeled as nonporous, due to higher viral transfer rates for nonporous fomites. For the subsequent single hand-to-orifice contact, the relevant routes of exposure for rotavirus, rhinovirus, and influenza A virus were considered. For rotavirus, it was assumed that a single contact to the mouth was made. Although rhinovirus and influenza A virus exposures can occur from contacts to the nose and eyes, microactivity data have shown that contacts to the nose are more frequent than to the eyes (30). Therefore, for rhinovirus and influenza A virus, it was assumed that a single contact to the nose was made. The following equations were used to calculate the transfer from fomite-to-hand and hand-to-mouth contacts for rotavirus, rhinovirus, and influenza A virus:

(1) $$C_{\text{hand}}=f_{12,\text{np}}\times C_{\text{surface}}\times C_{\text{adjust}}\times A_{\text{surface}}$$

(2) $$R_{\text{orifice}}=f_{23}\times C_{\text{hand}}$$

where *C*_hand_ is the number of viral particles on the hand, *f*_12,np_ is the fraction of virus transfer from a nonporous surface to the hand, *C*_surface_ is the virus concentration on a surface (genome copies per milliliter), *C*_adjust_ is the adjustment to convert *C*_surface_ to viral particles per square centimeter, *A*_surface_ is the surface area of hand-to-fomite contact (square centimeters), *R*_orifice_ is the viral dose (number of viral particles), and *f*_23_ is the fraction of virus transfer from hand to orifice.

For distributions of the parameters, see Table 5.

**TABLE 5:** Parameters for single fomite contact and 6-hour contact scenarios

Variable	Unit(s)	Distribution^*a*^	Reference(s) or source
*H*_porous_	Contacts/min	Log-normal (5.5, 1.5)	31, 34
*H*_nonporous_	Contacts/min	Log-normal (4.1, 1.6)	31, 34
*H*_nose_	Contacts/min	Log-normal (0.01, 66.7)	30, 31
*H*_mouth_	Contacts/min	Log-normal (0.18, 3.3)	31, 34
*H*_eyes_	Contacts/min	Log-normal (0.06, 3.3)	30, 31
*A*_nose_	cm^2^	Uniform (0.10, 10)	31
*A*_mouth_	cm^2^	Uniform (1, 41)	31, 35
*A*_eyes_	cm^2^	Uniform (0.10, 2)	31
*A*_hand_	cm^2^	Uniform (890, 1070)	31, 36
*A*_surface_	cm^2^	Point estimate 2	30
*f*_12,nonporous_	Fraction	Uniform (0.05, 0.22)	31, 37
*f*_12, porous_	Fraction	Uniform (0.0003, 0.0042)	31, 37
*f*_23_	Fraction	Point value 0.339	31, 38
*f*_21_	Fraction	Uniform (0.05, 0.22)	31, 37
FSA	Unitless	Log-normal (0.15, 1.2)	31, 39
Time	Min	Point value 360	This study
*C*_surface_	Genome copies/ml	Uniform (0, 3.4), exponential (0.00157, 3.4, 2.94 × 10^3^)	24, this study
*C*_adjust_	Viral particles/genome copies/100 cm^2^	Uniform [(1.533 × 10^−5^, 1.542 × 10^−1^)/100]	29, this study

a Log-normal (geometric mean, geometric standard deviation); uniform (minimum, maximum); exponential (rate, minimum, maximum).

Monte Carlo methods were used to include variability in the fraction of virus transfer for different contact types and in rotavirus, rhinovirus, and influenza A virus concentrations on surfaces. The model was run with 10,000 iterations. Predicted doses were then input into a beta-Poisson dose-response curve, with parameters specific to rotavirus, rhinovirus, and influenza A virus. The beta-Poisson curve was used, as it is the recommended dose-response curve for all three viruses by the QMRA Wiki (http://qmrawiki.canr.msu.edu/index.php/Quantitative_Microbial_Risk_Assessment_(QMRA)_Wiki):

(3) $$P_{\text{response}}=1-[1+\text{dose}\frac{2^{1/\alpha }-1}{N_{50}}{]}^{-\alpha }$$

### Case 2: modeling infection risk from 6 h of contact activity.

Six hours of exposure time was modeled because it was the time between seeding and surface sampling in the bacteriophage tracer study. To calculate the expected dose and infection risk, a steady state of virus concentration on hands was assumed. This method was used in a previous viral infection QMRA that argued steady state was appropriate because the rate at which virus leaves the hand is much lower than the overall exposure duration (31). This meant the concentration was constant during a single iteration for the full period modeled. An adapted version of the steady-state equation used by Beamer et al. (31) was used to calculate virus concentrations on hands at steady state:

(4) $$\bar{C_{\text{hands}}}=\frac{\sum_{j=1}^{j=m}\left(H_{\text{surface},j}\times f_{12,j}\right)\times C_{\text{surface}}\times C_{\text{adjust}}\times \text{FSA}}{\left(\sum_{j=1}^{j=m}H_{\text{surface},j}\right)\times f_{21}\times \text{FSA}+f_{23}\times \sum_{n=1}^{n=k}\left(H_{\text{oriface},n}\times A_{\text{oriface},n}\right)/A_{\text{hand}}}$$

where *C*_hands_ is the number of viral particles on the hand, *f*_12,np_ is the fraction of virus transfer from the surface to the hand, *C*_surface_ is the virus concentration on the surface (genome copies per milliliter), *C*_adjust_ is the adjustment to convert *C*_surface_ to viral particles per square centimeter, *A*_hand_ is the surface area of the hand (square centimeters), *H*_orifice,*n*_ is the number of hand-to-orifice contacts per minute, *H*_surface,*j*_ is the number of hand-to-surface contacts per minute, *A*_orifice,*n*_ is the surface area of hand-to-orifice contact, *f*_23_ is the fraction of virus transfer for hand-to-orifice contact, *f*_21_ is the fraction of virus transfer for hand-to-fomite contact, and FSA is the fraction of contact surface area per hand surface area.

For variable units and distributions, see Table 5. Viral decay was excluded, as enteric viruses have been shown to be persistent in environments on fomite surfaces, and respiratory viruses, such as rhinovirus and influenza A virus, have been shown to survive on surfaces for days (32, 33). Assuming no decay allows a conservative risk estimate. These hand concentrations (*C*_hands_) at steady state were then used to calculate the dose (31):

(5) $$R_{\text{orifice}}=f_{23}\times \overset{¯}{C_{\text{hands}}}\times \overset{n\;=\;k}{\underset{n\;=\;1}{\sum }}(H_{\text{orifice},n}\times A_{\text{orifice},n})\times \text{time}$$

Monte Carlo methods and iterations used in case 1 were also used in case 2. Additional distributions were included in case 2 to capture variability in the number of eye, nose, mouth, nonporous-surface, and porous-surface contacts per minute and the associated surface areas of contact. Predicted infection risks were calculated using the same dose-response curves used in case 1.

### Percent reduction calculations.

Median values were used to represent the central tendencies of the estimated infection risks. To investigate the effects of surface disinfection for those experiencing higher-than-average doses, the percent reduction in the 95th infection risk percentiles were quantified. Percent reductions in the median and 95th percentiles of infection risk were calculated by taking the difference between baseline-predicted and intervention-predicted risks of infection and then dividing by the baseline-predicted values:

(6) $$\%\text{Reduction}=\frac{\text{Baseline}-\text{Intervention}}{\text{Baseline}}\times 100\%$$

### Risk targets.

There are currently no risk targets for microbial infections in health care settings in the United States. The risk targets included in this study were 1/1,000, 1/10,000, 1/100,000, and 1/1,000,000. They were chosen so that the increase in percent reduction in surface concentrations needed to lower risk targets by 1 order of magnitude could be observed. Risk targets were chosen based on microbial drinking water standards and the standards used by Ryan et al. (15) to evaluate microbial health care infection risks.
